# (Pro)renin Receptor Is a Novel Independent Prognostic Marker in Invasive Urothelial Carcinoma of the Bladder

**DOI:** 10.3390/cancers13225642

**Published:** 2021-11-11

**Authors:** Gorka Larrinaga, Julio Calvete-Candenas, Jon Danel Solano-Iturri, Ana M. Martín, Angel Pueyo, Caroline E. Nunes-Xavier, Rafael Pulido, Juan F. Dorado, José I. López, Javier C. Angulo

**Affiliations:** 1 Department of Nursing, Faculty of Medicine and Nursing, University of the Basque Country (UPV/EHU), 48940 Leioa, Spain; 2 Department of Physiology, Faculty of Medicine and Nursing, University of the Basque Country (UPV/EHU), 48940 Leioa, Spain; 3 Biomarkers in Cancer Unit, Biocruces-Bizkaia Institute, 48903 Barakaldo, Spain; jondanel.solanoiturri@osakidetza.eus (J.D.S.-I.); CarolineElisabeth.NUNES-XAVIER@osakidetza.eus (C.E.N.-X.); rafael.pulidomurillo@osakidetza.eus (R.P.); joseignacio.lopez@osakidetza.eus (J.I.L.); 4 Service of Medical Oncology, University Hospital Puerta del Mar, 11009 Cádiz, Spain; jjcalvete@gmail.com; 5 Service of Pathology, Donostia University Hospital, 20014 San Sebastian, Spain; 6 Service of Pathology, University Hospital of Getafe, 28905 Madrid, Spain; amhita@salud.madrid.org; 7 Foundation for Biomedical Research and Innovation of University Hospitals Infanta Leonor and South-East, 28003 Madrid, Spain; angel.pueyo@salud.madrid.org; 8 Heath Science PhD Program, UCAM Universidad Católica San Antonio de Murcia, Guadalupe de Maciascoque, 30107 Murcia, Spain; 9 Department of Tumor Biology, Institute for Cancer Research, Oslo University Hospital Radiumhospitalet, 0310 Oslo, Norway; 10 Ikerbasque, The Basque Foundation for Science, 48011 Bilbao, Spain; 11 PeRTICA Statistical Solutions, 28906 Getafe, Spain; jfdorado@pertica.es; 12 Department of Pathology, Cruces University Hospital, 48903 Barakaldo, Spain; 13 Clinical Department, Faculty of Medical Sciences, European University of Madrid, 28005 Madrid, Spain; javier.angulo@universidadeuropea.es; 14 Department of Urology, University Hospital of Getafe, 28907 Madrid, Spain

**Keywords:** (pro)renin receptor, urothelial carcinoma, prognosis, biomarker

## Abstract

**Simple Summary:**

This is a novel description of (Pro)renin receptor (PRR) protein and its prognostic role in invasive urothelial cancer of the bladder. Using a tissue microarray, we investigated PRR expression and other immunohistochemical markers including p53, immune-checkpoint inhibition, and basal and luminal phenotypes in a series of patients with invasive urothelial carcinoma of the bladder treated with radical cystectomy. PRR expression is an independent prognostic marker and could be a potential target in urothelial carcinoma that should be further investigated.

**Abstract:**

(Pro)renin receptor (PRR) is being investigated in several malignancies as it activates pathogenic pathways that contribute to cell proliferation, immunosuppressive microenvironments, and acquisition of aggressive neoplastic phenotypes. Its implication in urothelial cancer (UC) has not been evaluated so far. We retrospectively evaluate the prognostic role of PRR expression in a series of patients with invasive UC treated with radical cystectomy and other clinical and histopathological parameters including p53, markers of immune-checkpoint inhibition, and basal and luminal phenotypes evaluated by tissue microarray. Cox regression analyses using stepwise selection evaluated candidate prognostic factors and disease-specific survival. PRR was expressed in 77.3% of the primary tumors and in 70% of positive lymph nodes. PRR expression correlated with age (*p* = 0.006) and was associated with lower preoperatively hemoglobin levels. No other statistical association was evidenced with clinical and pathological variables (gender, ASA score, Charlson comorbidity index, grade, pT, pN) or immunohistochemical expressions evaluated (CK20, GA-TA3, CK5/6, CD44, PD-L1, PD-1, B7-H3, VISTA, and p53). PRR expression in primary tumors was associated with worse survival (log-rank, *p* = 0.008). Cox regression revealed that PRR expression (HR 1.85, 95% CI 1.22–2.8), pT (HR 7.02, 95% CI 2.68–18.39), pN (HR 2.3, 95% CI 1.27–4.19), and p53 expression (HR 1.95, 95% CI 1.1–3.45) were independent prognostic factors in this series. In conclusion, we describe PRR protein and its prognostic role in invasive UC for the first time. Likely mechanisms involved are MAPK/ERK activation, Wnt/β-catenin signaling, and v-ATPAse function.

## 1. Introduction

Urinary bladder cancer is the most prevalent genitourinary malignancy in industrialized countries, with more than half a million new diagnoses and approximately 200,000 deaths worldwide per year [1]. Age-standardized incidence rate exceeds 16 new cases per 100,000 males in Europe, the United States, and Canada [2]. Despite the heterogeneity of urothelial carcinoma (UC) of the bladder, a vast majority of the cases are transitional cell carcinoma, and the most important clinico-pathological parameter to define prognosis, depth of tumor invasion within the bladder wall, has been established decades ago [3]. Definitely, there is a need for novel biomarkers to improve the utility of prediction tools for bladder cancer in an era in which novel immunotherapy is developing [4].

Research, investments, and advances in bladder cancer have been limited compared to other cancers. Immunotherapies with immune checkpoint inhibitors targeting the programmed death-1 (PD-1) receptor or its ligand (PD-L1) and cytotoxic T-lymphocyte antigen 4 (CTLA-4) have demonstrated a role after failure of cisplatin-based chemotherapy, but the majority of the patients do not respond to this strategy [5], and biomarkers to predict patients who could benefit from checkpoint targeting therapy are presently lacking [6]. Additionally, the role of chemokines as modulators of tumor angiogenesis and their potential as therapeutic targets is currently under investigation [7,8].

Another major recent advance in UC is the definition of molecular subtypes based on genomic expression patters (neural-like, HER2-like, papillary-like, luminal-like, mesenchymal-like, and squamous-cell carcinoma-like). Different risk of progression was confirmed and, more interestingly, distinct pathways and likely targets are involved for each subtype [9]. We are far from being able to define the therapeutic implications of these findings yet, but a more simplified approach based on the distinction of basal (CK5/6, CD44) and luminal (GATA3, CK20) phenotypes is more practical for diagnostic and prognostic purposes as it can be identified by signature immunohistochemical (IHC) markers [10,11].

The search for new and better markers is far from being closed. A recently proposed novel biomarker and candidate therapeutic target for several malignancies is the (pro)renin receptor (PRR). As far as we know, its potential has not been investigated in UC. PRR is a single transmembrane protein encoded by the ATP6AP2 gene located on the X chromo-some [12]. It participates in a range of normal and disease processes including vacuolar ATPase (V-ATPase) function and the Wnt/β-catenin signaling pathway [13]. PRR was initially investigated as part of the renin–angiotensin system (RAS) for its role in the activation of the MAPK/ERK pathway through the binding of RAS to its ligands renin and/or prorenin [14]. Very recently, the prognostic role of PRR immunohistochemical expression has been confirmed in colorectal, breast, prostate, pancreatic, and renal cancers [13,15,16,17,18,19,20]. Additionally, PRR has been used as a molecular target for cancer diagnosis using single-photon emission computed tomography [21], and a new therapeutic strategy based on monoclonal antibodies against PRR is currently being investigated in pancreatic neo-plasia [22].

We aim to evaluate the role of PRR expression in a sample of patients with invasive UC treated with radical cystectomy and investigate its potential role as prognostic marker. The association between PRR expression and other clinical, histopathological, and immunohistochemical markers (p53, PD-1, PD-L1, B7-H3, VISTA, CK5/6, CD44, CK20, and GAT3) has been investigated with the intention to evaluate the interrelationship between PRR and these variables and better assess whether PRR behaves as an independent prognosticator.

## 2. Materials and Methods

### 2.1. Patients and Samples

This is a retrospective study carried out on a series of 119 patients with non-metastatic UC of the bladder. All cases were high-grade transitional cell carcinoma treated with radical cystectomy including lymph node dissection between 2000 and 2015. All the registers were included in a database with the approval of the Institutional Review Board (IRB, A06/16). Patients with positive lymph nodes and locally advanced disease were offered cisplatinum-based adjuvant chemotherapy (gemcitabine plus cisplatin or mitomycin, vincristine, adriamycin plus cisplatin). Neoadjuvant chemotherapy was not used during the period investigated. All patients were followed up until death or until the data were censored. At that time, 47 patients were alive, 55 were dead of disease, and 17 died of other causes. All living subjects were informed about the potential use for research of their surgically resected tissues and accepted this eventuality by signing a specific IRB-approved document.

The primary endpoint of the study was the evaluation of cancer-specific mortality. Clinical parameters before cystectomy (age, ASA score, Charlson comorbidity index, preoperative hemoglobin, and transfusion) were also evaluated. Two pathologists collected representative formalin-fixed and paraffin embedded (FFPE) tissue blocks for both primary tumor (*n* = 119) and lymph nodes (*n* = 30) and a third pathologist (JIL) reviewed all the specimens; confirmed histological type, histological grade, and tumor stage (AJCC/TNM 2017); and performed immunohistochemical evaluation.

### 2.2. Tissue Microarray Construction and Immunohistochemical Staining

Tissue microarrays (TMA) were performed selecting tissue samples with abundant tumor tissue without artifact, when possible. For each case, two tumor samples (2.5 mm in diameter) were transferred from the original paraffin block to the recipient TMA block. Whenever allowed by the size of the tumor seed within lymph nodes, two additional samples were obtained from lymph node metastases and transferred following the same process. Consecutive 4 µm sections were performed from TMA blocks, and the first one was stained with hematoxylin-eosin to verify the proper construction of the blocks and that representative material was present in all cases.

An extensive immunohistochemical (IHC) study was carried out with PRR, GATA3, CK20, CK5/6, CD44, PD-L1, PD-1, B7-H3, VISTA, and p53 antibodies. PRR antibody (HPA003156; Sigma-Aldrich at 1/50 dilution, cytoplasmic staining) was evaluated in tu-mor cells. Cytoplasmic staining was evaluated as negative, weak, or intense following previously described scores [18,20]. GATA3 (Ventana, ref. L50–823, ready-to-use, nuclear staining), CK20 (Ventana ref. SP-33, ready-to-use, cytoplasmic staining), CK5/6 (Ventana ref. D5/16B4, ready-to-use, cytoplasmic staining), CD44 (Ventana ref. SP-37, ready-to-use, cytoplasmic staining), p53 (Ventana, ref. DO-7, ready-to-use, nuclear staining), B7-H3 (R&D, ref. AF1027, dilu-tion 1:2000), and VISTA (Cell Signaling, ref. 64953, dilution 1:100) antibodies were also evaluated in tumor cells. PD-1 (Ventana, ref. NAT105, ready-to-use) was evaluated in intratumor inflammatory cells. These antibodies were evaluated as positive or negative, as usually performed in the clinical practice. Finally, PD-L1 (Ventana ref. SP-142, ready-to-use, cytoplasmic staining) was considered positive with a staining of ≥5% of cells, as recommended by the manufacturer. Automated immunostaining (EnVision FLEX, Dako Au-tostainer Plus; Dako, Glostrup, Denmark and BenchMark Ultra, Ventana Medical Sys-tems, Tucson, AZ, USA) followed routine methods. Tris-EDTA was used for antigen retrieval. Negative controls were slides not exposed to the primary antibody, and these were incubated in PBS and then processed under the same conditions as the test slides. The analysis was performed using a Nikon Eclipse 80i Microscope (Tokyo, Japan).

### 2.3. Statistical Analysis

SPSS^®^ 24.0 Software was used for the statistical analysis. A Kolmogorov–Smirnov test was applied to determine whether the numbers followed or not a normal distribution. Based on this information, data were analyzed with non-parametric tests. We performed a Spearman Rho test to evaluate the correlation between PRR expression and patient age. The Chi-square (χ^2^) test was used to analyze the categorical PRR expression (negative, weak, or moderate/strong) in UC tissues (primary tumors and node metastases), the association between PRR expression and patients’ gender, the association with pathological variables of cancer aggressiveness, and the association between this protein and biomarkers of luminal and basal phenotypes of UC, immune checkpoints, and p53.

Kaplan–Meier curves and log-rank test were performed to evaluate the association between the expression of PRR and cancer-specific survival of UC patients. Groups were created by cut-off points based on categorical PRR expression in tumor tissue (negative, weak, or moderate/strong). Finally, to evaluate the independent effects of PRR expression and clinical and pathological variables on cancer-specific survival, univariate and multivariate analyses was performed using Cox proportional hazards regression model with a threshold entry *p* = 0.1 and a threshold stay *p* = 0.05.

## 3. Results

The main clinical and histopathological characteristics of the series analyzed are shown in Table 1. The mean follow-up since cystectomy was 53.1 ± 48.8 (range 3–193) months. The mean age of the patients at the time of radical cystectomy was 68.1 ± 9.25 (range 44–89) years. Globally, 24.4% of the patients received adjuvant systemic chemotherapy. Histopathological staging revealed locally advanced disease, including perivesical infiltration (pT3) or invasion of neighboring organs (pT4), in 39.7% of the patients and positive nodal disease (pN1–3) in 40.5%. Median preoperative hemoglobin was 13.2 ± 2.1 (range 7.8–17.3) g/dl, and transfusion rate, including intra and postoperative transfusion, was 32.2%

**Figure 1:**
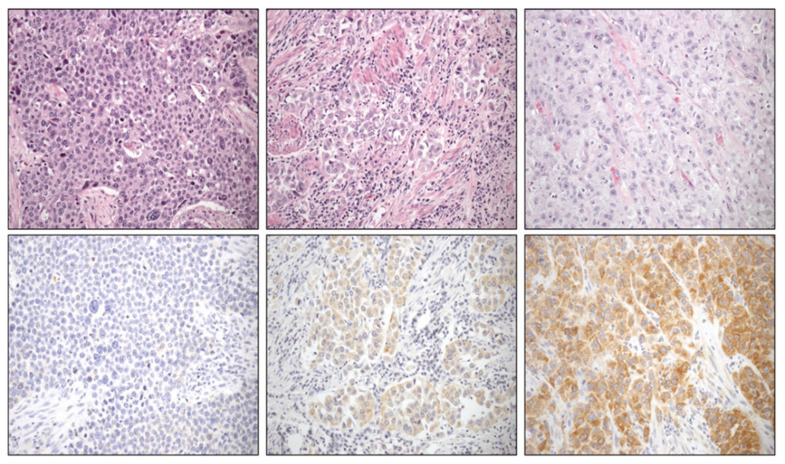
High-grade urothelial carcinomas with their respective prorenin immunostaining (lower row) quantified as absent (**left**), weak (**middle**), and intense (**right**) (original magnification in all cases × 250).

PRR was expressed in 77.3% of the primary tumors and in 70% of invaded lymph nodes, always restricted to epithelial tumor cells (Figure 1). In terms of immunostaining intensity, 54.6% of primary tumors showed weak expression and 22.7% showed intense staining. There was not any significant difference with PRR expression in UC infiltrating lymph nodes, which was weak in 50% of cases and intense in 20% (Chi-square, *p* = 0.703).

PRR expression in UC tissues positively correlated with patient age (*r* = 0.251, *p* = 0.006) but not with gender (Chi-square test, *p* = 0.167). Table 2 describes the association between PRR expression in primary UCs and clinical and pathological variables.

This protein was similarly expressed in high-grade UC with different levels of bladder wall invasion (pT) and in tumors with or without nodal invasion (pN). That is, patients with extravesical disease or lymph node metastasis did not have a different pattern of PRR immunostaining. Additionally, associated carcinoma in situ did not follow a different pattern.

From a clinical perspective, ASA physical status classification system and Charlson comorbidity index were neither associated with different expression of PRR in UC tissues. However, lower preoperative hemoglobin levels (<13 mg/dL) were significantly associated with PRR expression. Patients who received cisplatinum-based adjuvant chemotherapy did not express higher PRR than patients treated with cystectomy alone.

We also analyzed the association between PRR and several biomarkers with prognostic and therapeutic implications, including luminal (CK20 or GATA3) and basal (CK5/6 or CD44) UC phenotypes, immune checkpoints (PD-L1, PD-1, B7-H3, and VISTA) and p53. PRR was not significantly associated with any of the mentioned immunohistochemical markers, which confirms the independent mechanism and effect of PRR (Table 3). Additionally, the combination of both luminal markers (CK20 and GATA3) and both basal phenotype markers investigated (CK5/6 and CD44) is not associated with PRR expression (Chi-square test, *p* = 0.896 and *p* = 0.905; respectively).

**Figure 2:**
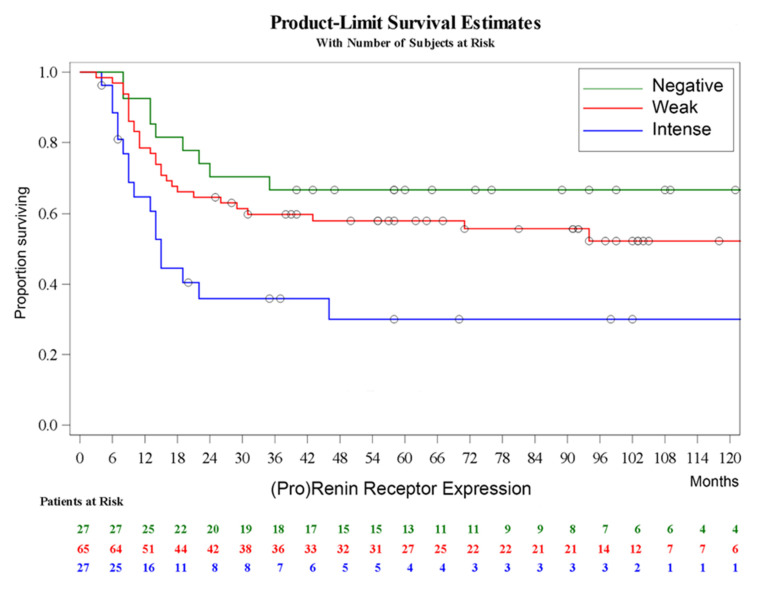
Kaplan–Meier curve of disease-specific survival according to the immunohistochemical expression of prorenin receptor (absent, weak, and intense) in patients with urinary bladder cancer treated by cystectomy; log-rank, *p* = 0.008.

The Kaplan–Meier curves and log-rank test showed that PRR expression in primary tumors significantly predicts cancer-specific survival of UC patients (log-rank, *p* = 0.008). Intense IHC positivity follows the worst prognosis while weak IHC staining follows an intermediate course between intense and absent IHC staining (Figure 2). On the other hand, evaluation of disease-specific survival according to the immunohistochemical expression of other likely individual tumor markers evaluated in this series with likely influence on prognosis is presented in Figure S1, Supplementary Data. Markers of basal or luminal phenotypes did not predict prognosis when assessed individually. Similarly, markers of immune checkpoint inhibition could not be elected as prognostic markers in this series (Figure S1(A1–A9)).

Before evaluating the independent effects of PRR expression on patients’ survival by a multivariate Cox regression, we performed univariate analyses with the variables evaluated. Since a significant positive correlation between PRR and age was observed, we first tested the effect of age in survival. The median value of the age of patients was selected as a cut-off value, and it was observed that it was significantly associated with cancer-specific survival. We also included other clinical variables (ASA score, Charlson comorbidity index, preoperative hemoglobin, and the need of adjuvant chemotherapy), pathological variables (pT, pTIS, and pN), and the mentioned biomarkers of UC phenotypes, immune checkpoints, and cell cycle.

In the univariate analysis, patient age was the only statistically significant clinical variable. Among the IHC markers, only PRR appeared overtly significant, and p53 expression almost reached it, when considered individually. Among the classical histopathological variables, both pT (local invasion level) and pN (status of lymph nodes) were statistically significant.

The multivariate analysis demonstrated that PRR expression in primary UC, together with pT, pN, and p53 protein, is an independent prognostic factor. The final step of the Wald method of the multivariate analysis also selected CK5/6 expression, although it did not reach statistical significance (Table 4).

## 4. Discussion

(Pro)renin receptor (PRR), also known as ATP6AP2, is a relatively new discovered component of the renin angiotensin system (RAS) that activates prorenin enzyme and enhances the activity of renin enzyme [14]. This multifunctional protein is involved in many RAS-dependent and independent pathways and has been very recently suggested to play an important role in neoplasia [13]. RAS itself has been widely associated with the progression of different types of cancer [23]. Epidemiological and translational data [24,25] strongly suggest an important role of RAS in the main hallmarks of cancer [13]. Additionally, it has been suggested that targeting the RAS system using RAS inhibitors may have beneficial effects in a broad range of malignancies, as it could reduce the side effects of immunotherapy and also improve response to treatment with immune checkpoint inhibitors and prognosis of certain tumors [26,27].

It has been recently determined that PRR has an essential role for Wnt signaling pathway activation through the Frizzled (Fz) receptor [28]. The Wnt pathway is involved in bladder cancer progression by triggering processes such as proliferation or cell motility [29]. Additionally, the Wnt/β-catenin signaling pathway is very important to enhance epithelial–mesenchymal transition in human bladder cancer [30]. Based on this evidence, the potential tumorigenic action of PRR observed in our immunohistochemical analysis could be explained by the fact that the overexpression of PRR may trigger an over-activation of the Wnt signaling pathway and, hence, lead to urothelial cancer stem cell self-renewal and progression of UC and chemoresistance [29].

The truncated form of PRR is an accessory protein of the v-ATPase and has a role in the acidification of intracellular compartments and the regulation of autophagy, a process required to maintain cellular environmental homeostasis through the degradation and recycling of damaged cytoplasmic components and organelles [31]. Autophagy contributes to the maintenance of UC cell survival [32], and this could be another mechanism involved in the diminished survival of patients revealing intense IHC expression of PRR in this series of non-metastatic UC. However, as far as we know, there is no information regarding a potential consequence of the over-expression of PRR on the functions related to v-ATPase. Hence, additional investigation is required to investigate the potential effects of PRR over-expression in the framework of v-ATPAse.

One of the most interesting aspects of the evaluation of PRR is that IHC evaluation can be defined in a simple model as negative, weak, and intense. Of course, our interpretation should be evaluated in future studies with other tumor samples, and also desirably in a prospective setting. However, PRR IHC evaluation is likely to be reproducible both from the laboratory process and pathologist interpretation, taking into account that similar findings have been reported by our group for different tumors [18,20].

Other simple IHC markers have been validated in our array sample, mainly CK5/6 and p53. In fact, CK5/6 expression in UC without squamous differentiation has been recently defined as an independent prognostic biomarker [33]. Although extensively tested, results regarding p53 expression are conflicting as surrogate marker for p53 mutation. Most studies report that this molecule is valuable to determine prognosis, although problems related to antibody selection, lack of standardization, and different cut-off values used have yielded equivocal results [34]. Although it has been investigated for decades, there is not sufficient evidence to conclude whether changes in p53 can be used as a precise marker of prognosis in UC [35]. In our study, IHC expression of p53 and PRR behave as independent predictors of UC specific survival. Interestingly and in order to approach an independent value in Cox regression, IHC expression of CK5/6 is a marker of marginal importance compared to PRR. The prognostic value of basal-type markers could be enhanced when the co-expression of stromal marker fibroblast activation protein (FAP) is evaluated [11,36]. Other phenotype markers of UC and markers of immune checkpoint inhibition evaluated did not predict prognosis in this series, and that can be a consequence of the difficulties involved in the interpretation of immune-checkpoint inhibition based only on PD-1 and PD-L1 IHC results, as has also been revealed in renal cell carcinoma [37]

Noticeably, the value of PRR as a new tissular prognostic factor in UC does not de-pend on classical histopathological parameters, such as tumor grade, parietal depth of tumor invasion, or lymph node infiltration, and this is an observation of utmost im-portance. Additionally, we demonstrate that PRR expression is not associated with the expression of basal (CK 5/6, CD44) or luminal phenotype markers (CK20, GATA3) or to p53. Additionally, immune checkpoint inhibition markers (PD-L1, PD-1, B7H3, or VISTA) appear completely unrelated to PRR expression. These data sustain the independent prognostic value of PRR expression in patients with invasive UC of the bladder. Only the association between PRR and p53 immunostaining approaches statistical significance. This is very interesting because, in the Cox proportional-hazards model regression, both PRR and p53 staining are independent predictors, together with tumor depth of invasion (pT cat-egory) and lymph node invasion (pN category). The prognostic value of CK5/6 expression and also other clinical variables such as patient age and preoperative hemoglobin are in the limit of statistical significance in univariate analysis but lose their prognostic value in the multivariate regression model. Regarding the association we observed between negative PRR expression and higher Hb levels (Table 2), it has already been suggested that PRR is expressed in erythroblastic cells and also that it may contribute to the homeostatic control of erythropoiesis [38].

The main limitations of our investigation stand in its retrospective nature and also in a relative limited number of patients. However, under the light of our analysis, PRR is a strong independent predictor of diminished bladder-cancer-specific survival, and this is the first study describing this protein and its role in invasive UC. Functional studies should also be performed to understand the basic role of PRR in bladder cancer biology and how this novel marker influences cancer progression and aggressiveness in UC. According to investigation performed in other tumor models, likely mechanisms are MAPK/ERK activation, Wnt/β-catenin signaling, and v-ATPAse function [39]. The role of PRR in cancer development and progression can also be supported by big data analysis from The Cancer Genome Atlas (TCGA) and Genotype-Tissue Expression (GTEx) data-bases in several malignancies [13], although further specific investigation is needed in UC. This clinical study opens the perspective to investigate development of novel therapies for UC based on neutralizing anti-PRR molecular antibodies to suppress Wnt/β-catenin signaling [22].

## 5. Conclusions

Despite the plethora of UC prognostic markers identified in the last decades, there is still a role for definition of new tumor markers in this prevalent and serious malignancy. The prognostic role of PRR protein in different neoplasia is being currently investigated. We confirm this novel tumor marker could be of primary importance in UC, as different patterns of IHC expression of PRR define disease-specific survival in UC treated with radical cystectomy. What is remarkable is that this prognostic role seems to be independent of classical histopathological features including the depth of bladder wall invasion and presence of lymph node metastases. Additionally, immunohistochemical expression of PRR seems to predict disease-specific prognosis in UC of the bladder much better than other markers, including luminal (CK20 and GATA3) and basal (CK5/6 and CD44) UC phenotypes, immune checkpoints (PD-L1, PD-1, B7-H3, and VISTA) and p53. In summary, evidence is provided that PRR IHC expression has a prognostic role in patients with muscle-invasive bladder cancer treated with radical cystectomy.

## Figures and Tables

**Table 1:** Characteristics of the series (*n* = 119); * Mean ± SD.

	*n* (%)
*Sex*	
Male	116 (97.5)
Female	3 (2.5)
*Age*, years *	68.1 ± 9.25
*pT category*	
pT1	13 (10.9)
pT2	27 (22.7)
pT3	51 (42.9)
pT4	28 (13.5)
*pN category*	
pN0	72 (60.5)
pN1	21 (17.6)
pN2	25 (21)
pN3	1 (0.8)
*Histological grade (WHO)*	
G2	4 (3.4)
G3	115 (96.6)
*Associated carcinoma in situ*	
Yes	41 (34.5)
No	78 (65.5)
*Preoperative Hb*, g/dL *	13.2 ± 2.1
*Adjuvant chemotherapy*	
Yes	29 (24.4)
No	90 (75.6)
*Bladder cancer mortality*	
Yes	55 (46.2)
No	64 (53.8)

WHO, world Health Organization; Hb, hemoglobin.

**Table 2:** Association between PRR expression and clinico-pathological characteristics.

	PRR Immunostaining			
Variables	Negative (%)	Weak (%)	Intense (%)	*p* Value
**Histopathological features**				
*Grade (WHO)*				
G2 (*n* = 4)	25	75	0	0.532
G3 (*n* = 115)	22.6	53.9	23.5	
*Local invasion (pT)*				
pT1–pT2 (*n* = 40)	15	65	20	0.229
pT3–pT4 (*n* = 79)	26.6	49.4	24.1	
*pTis*				
No (*n* = 78)	28.2	50	21.8	0.135
Yes (*n* = 47)	12.2	63.4	24.4	
*Node invasion (pN)*				
No (*n* = 72)	25	56.9	18.1	0.311
Yes (*n* = 47)	19.1	51.1	29.8	
**Clinical variables**				
*ASA score*				
I–II (*n* = 85)	25.9	52.9	21.2	0.408
>III (*n* = 34)	14.7	58.8	26.5	
*Charlson comorbidity index*				
1–2 (*n* = 44)	27.3	52.3	20.5	0.647
>3 (*n* = 75)	20	56	24	
*Preoperative serum Hb*				
<13 mg/dl	11.8	66.7	21.6	0.029
≥13 mg/dl	30.9	45.6	23.5	
*Adjuvant chemotherapy*				
No (*n* = 90)	23.3	56.7	20	0.466
Yes (*n* = 29)	20.7	48.3	31	

WHO, World Health Organization; ASA, American Society of Anesthesiologists; Hb, hemoglobin.

**Table 3:** Association between PRR expression and other immunohistochemical markers.

	PRR Immunostaining			
Variables	Negative (%)	Weak (%)	Intense (%)	*p* Value
**Luminal phenotype**				
*CK20*				
Negative (*n* = 73)	24.7	53.4	21.9	0.807
Positive (*n* = 41)	19.5	58.5	22	
*GATA3*				
Negative (*n* = 32)	28.1	56.3	15.6	0.41
Positive (*n* = 84)	20.2	53.6	26.2	
**Basal Phenotype**				
*CK5/6*				
Negative (*n* = 68)	20.6	58.8	20.6	0.782
Positive (*n* = 44)	25	52.3	22.7	
*CD44*				
Negative (*n* = 54)	24.1	50	25.9	0.672
Positive (*n* = 60)	20	58.3	21.7	
**Immune checkpoints**				
*PD-L1*				
Negative (*n* = 74)	20.3	58.1	21.6	0.807
Positive (*n* = 39)	25.6	53.8	20.5	
*PD-1*				
Negative (*n* = 46)	19.6	60.9	19.6	0.609
Positive (*n* = 68)	25	51.5	23.5	
*B7-H3*				
Negative (*n* = 46)	21.7	52.2	26.1	0.833
Positive (*n* = 66)	22.7	56.1	21.2	
*VISTA*				
Negative (*n* = 32)	18.8	56.3	25	0.858
Positive (*n* = 82)	23.2	54.9	22	
**Cell-cycle regulation**				
*p53*				
Negative (*n* = 51)	33.3	47.1	19.6	0.054
Positive (*n* = 63)	14.3	61.9	23.8	

WHO, World Health Organization; ASA, American Society of Anesthesiologists; Hb, hemoglobin.

**Table 4:** Cox regression model for cancer-specific survival prediction stepwise model with *p* = 0.1 in this series; statistically significant values highlighted in bold.

Variables	*p* Value	Exp (B)	Lower C.I.	Upper C.I.
**Univariate**				
*Age* > 68 vs. ≤68 years	**0.021**	1.832	1.094	3.066
*Local invasion* pT3–4 vs. pT1–2	**0.2 × 10^6^**	7.918	3.391	18.489
*Lymph Node invasion* yes vs. no	**0.2 × 10^7^**	4.062	2.383	6.925
*Tumor grade (WHO)* G3 vs. G2	0.272	21.758	1.094	5.29 × 10^3^
*Carcinoma* in situ present vs. absent	0.310	1.311	0.777	2.213
*Preoperative Hb* > 13 vs. ≤13 g/dL	0.135	0.677	0.406	1.13
*ASA score* ≥ III vs. I–II	0.146	1.492	0.87	2.561
*Charlson* > 2 vs. ≤2	0.136	1.536	0.874	2.701
*Adjuvant chemotherapy* yes vs. no	0.283	1.362	0.775	2.392
*CK20* positive vs. negative	0.798	0.931	0.537	1.613
*GATA3* positive vs. negative	0.258	0.724	0.414	1.267
*CK5/6* positive vs. negative	0.123	1.513	0.894	2.562
*CD44* positive vs. negative	0.443	1.228	0.727	2.072
*PD-L1* positive vs. negative	0.993	0.998	0.568	1.753
*PD-1* positive vs. negative	0.384	0.79	0.465	1.343
*B7-H3* positive vs. negative	0.879	0.96	0.569	1.622
*VISTA* positive vs. negative	0.873	0.954	0.539	1.692
*p53* positive vs. negative	0.061	1.692	0.976	2.933
*PRR* intense vs. weak vs. negative	**0.005**	1.808	1.195	2.734
**Multivariate**				
*Local invasion* pT3–4 vs. pT1–2	**0.000**	7.016	2.676	18.395
*Lymh node invasion* yes vs. no	**0.006**	2.297	1.27	4.187
*p53* positive vs. negative	**0.022**	1.947	1.099	3.448
*PRR* intense vs. weak vs. negative	**0.004**	1.851	1.222	2.802

WHO, World Health Organization; ASA, American Society of Anesthesiologists.

## Data Availability

Full data will be available from the corresponding author upon reasonable request.

## Supplementary Materials

The following are available online at https://www.mdpi.com/article/10.3390/cancers13225642/s1, Figure S1: Kaplan–Meier curve of disease-specific survival according to the immunohistochemical expression of markers of including luminal CK20 (log-rank, *p* = 0.795; A1), GATA3 (log-rank, *p* = 0.25; A2), CK5/6 (log-rank, *p* = 0.115; A3), CD44 (log-rank, *p* = 0.436; A4), PD-L1 (log-rank, *p* = 0.993; A5), PD-1 (log-rank, *p* = 0.377; A6), B7-H3 (log-rank, *p* = 0.877; A7), VISTA (log-rank, *p* = 0.872; A8), and p53 (log-rank, *p* = 0.055; A9) in patients with urinary bladder cancer treated by cystectomy.
