# Supplementary figures and images for: (Pro)renin Receptor Is a Novel Independent Prognostic Marker in Invasive Urothelial Carcinoma of the Bladder
Source: Cancers (Basel). 2021 Nov 11;13(22):5642. doi: 10.3390/cancers13225642 (PMC8616163; doi:10.3390/cancers13225642)

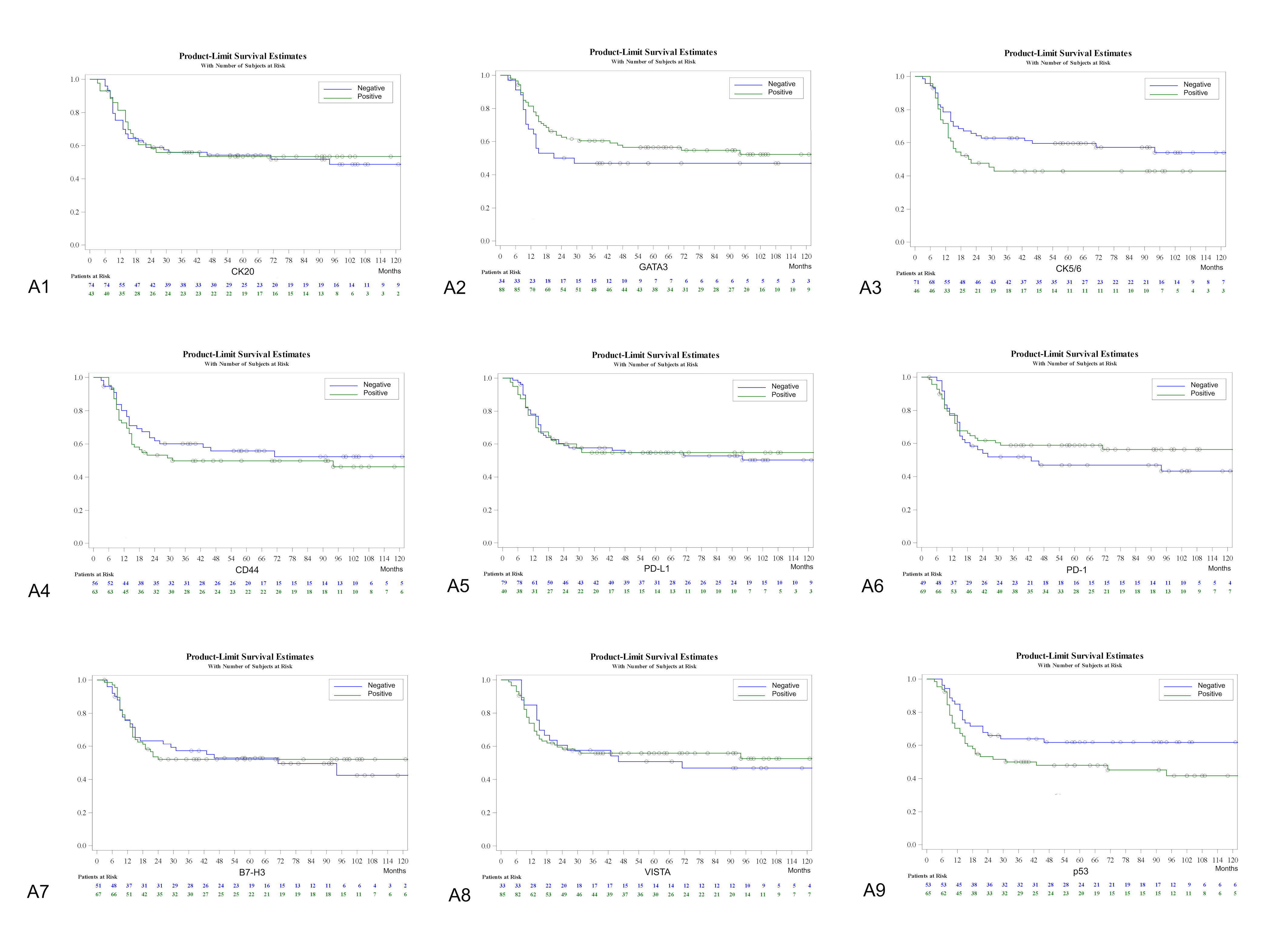

Supplement: Supplementary file 1 [file cancers-13-05642-s001.zip › cancers-1425171-supplementary.jpg]
